# SPOCK2 and SPRED1 function downstream of EZH2 to impede the malignant progression of lung adenocarcinoma in vitro and in vivo

**DOI:** 10.1007/s13577-023-00855-0

**Published:** 2023-01-11

**Authors:** Yang Liu, Xiaoxi Fan, Changrui Jiang, Shun Xu

**Affiliations:** Department of Thoracic Surgery, The First Hospital of China Medical University, No. 155, Nanjing North Street, Shenyang, Liaoning China

**Keywords:** EZH2, Lung adenocarcinoma, SPOCK2, SPRED1, Malignant progression

## Abstract

Enhancer of zeste homolog 2 (EZH2) is an important epigenetic regulator, and is associated with the malignant progression of lung cancer. However, the mechanisms of EZH2 on lung adenocarcinoma (LUAD) remain unclear. The relationship between EZH2 and SPOCK2 or SPRED1 was confirmed by dual-luciferase reporter assay. The Cancer Genome Atlas (TCGA) and Gene Expression Omnibus (GEO) databases were analyzed to examine the expression of SPOCK2 and SPRED1 and their prognostic values of LUAD. The effects of SPOCK2 and SPRED1 on the biological characters of LUAD cells were identified on functional assays in vitro and in vivo. Our results showed that EZH2 suppressed the expression and transcriptional activity of SPOCK2 and SPRED1, and these effects were reversed by the EZH2 inhibitor, Tazemetostat. SPOCK2 and SPRED1 were expressed at low levels in LUAD patients, and a high expression level of SPOCK2 or SPRED1 predicted better survival. Moreover, overexpression of SPOCK2 or SPRED1 could inhibit tumoral proliferation, migration ratio, and invasion activity in vitro as well as retard tumor growth in vivo. However, EZH2 elevation could rescue these impacts and accelerate LUAD progression. Our findings reveal that SPOCK2 and SPRED1 are epigenetically suppressed by EZH2 and may act as novel regulators to inhibit the proliferation, migration, and invasion of LUAD cells.

## Introduction

Lung cancer is one of the most serious malignant tumors threatening human life [1]. Worldwide, lung cancer has the highest mortality rate of all cancers, with an estimated 1.8 million deaths (18%) [2]. None small-cell lung carcinoma (NSCLC) is the major subtype of lung cancer, of which lung adenocarcinoma (LUAD) is a common histologic subtype of NSCLC, accounting for 40–80% of all NSCLC diagnoses [3]. Worryingly, LUAD is common in women and non-smokers and is gradually increasing in these individuals [4]. The 5-year survival rate of LUAD after complete invasion is usually only 15%, despite advancements in diagnostic techniques and molecularly targeted therapies [5, 6]. Thus, more molecular-based tools are needed to enhance the survival and treatment prediction for patients.

Enhancer of zeste homolog 2 (EZH2) is a histone methyltransferase, an enzymatic catalytic subunit of polycomb repressive complex 2 (PRC2) [7]. EZH2 is an important epigenetic regulator that represses transcription, it regulates the transcription of downstream genes by trimethylation of H3 on lysine 27 (H3K27me3) [8]. H3K27 methylation is associated with the inhibition of gene expression and is considered a critical event during the cell development and the progress of diseases [9]. Recent studies have revealed a critical role of EZH2 in proliferation, metastasis, drug resistance and immune regulation of tumor cells. For instance, Chu et al. has found that EZH2 promotes the growth and metastasis of breast cancer cells [10]. Furthermore, inhibition of EZH2 significantly enhanced SLFN11 expression and improves the chemosensitivity of lung cancer cells, thus preventing the emergence of acquired resistance [11]. Undoubtedly, EZH2 has multiple functions in tumor biology and new mechanisms remain to be discovered.

The secreted protein acidic and cysteine-rich gene in combination with osteonectin (SPARC), cwcv and kazal-like domains proteoglycan 2 (SPOCK2), is a member of the SPOCK family. Sprouty-related Ena/VASP homology 1 (EVH1) domain containing 1 (SPRED1) is a member of the SPRED family of proteins. Several studies have indicated that SPOCK2 and SPRED1 expression were elevated in EZH2 knockout cells [12, 13]. These evidences suggest that EZH2 may have regulatory roles on SPOCK2 and SPRED1. However, their potential functions and underlying mechanisms in LUAD are still unclear.

Therefore, the aim of this study was to explore the mechanisms of EZH2 targeting SPOCK2 and SPRED1 and the functional impacts of SPOCK2 and SPRED1 in LUAD. First, the regulatory mode of EZH2 on SPOCK2 or SPRED1 was investigated. Second, the expression of SPOCK2 and SPRED1 and their prognostic roles in LUAD patients were evaluated in The Cancer Genome Atlas (TCGA) database. Third, we analyzed the abilities of proliferation, migration and invasion in LUAD cells to gain further insights into the biological functions of SPOCK2 and SPRED1 in LUAD. We also assessed whether EZH2 is involved in regulating the effects of SPOCK2 and SPRED1 on the malignant behaviors of LUAD. Finally, nude mice were used to verify the functions of SPOCK2 and SPRED1 on LUAD in vivo.

## Materials and methods

### Cell culture

Human lung cancer cell lines A549 and HCC827 were procured from Procell Life Science &Technology Co., Ltd. (CL-0016, China) and iCell Bioscience Inc (iCell-h068, China). A549 cells were cultured in Ham’s F-12 K (Procell, China) with 10% fetal bovine serum (FBS, Tianhang, China). HCC827 cells were cultured in RPMI-1640 (Solarbio, China) supplemented with 10% FBS. All cell lines were placed in a humidified incubator containing 5% CO_2_ at 37 °C.

### Transfection of plasmids

EZH2, SPOCK2, SPRED1 overexpression plasmid or pcDNA3.1 control plasmid were transfected in cells by using Lipofectamine 3000 reagent (Invitrogen, US). Twenty-four hours after transfection, the cells were harvested for western blot analysis. The stable cell lines were obtained after 1–2 weeks of G418 screening.

### EZH2 inhibitor treatment

To evaluate the effect of EZH2 inhibition on SPOCK2, SPRED1, EZH2, and H3K27me3, A549 cells were treated with different concentrations of tazemetostat (inhibitor for EZH2; Shanghai yuanye Bio-Technology, China) for 72 h, then analyzed by quantitative RT-PCR (qRT-PCR) and western blotting.

### Xenograft study

Nude mice between 6 and 8 weeks of age were used for this study. The mice were housed under standard laboratory conditions in 12 h dark/light cycle at an ambient temperature of 25 ± 1 °C with 45–55% humidity. They were provided continuous food and water supply.

Stably transfected A549 cells (2 × 10^6^ cells) were subcutaneously injected into the right axilla of nude mice. Tumor growth was monitored by measurement of tumor volumes every 4 days. After 28 days, tumors were harvested, photographed, and used for further experiments.

### Bioinformatics analysis

The expression of SPOCK2 and SPRED1 in LUAD were evaluated using the UALCAN (http://ualcan.path.uab) online analysis software [14]. Human Protein Atlas (https://www.proteinatlas.org/) was utilized for survival analysis of SPOCK2 and SPRED1 TCGA LUAD database [15]. The best high and low expression cut-off values were 9.68 for SPOCK2 and 7.73 for SPRED1.

### Cell counting Kit-8 (CCK-8) assay

The transfected cells were seeded in 96-well plates (5 × 10^3^ cells/well). CCK-8 reagent (10μL; Solarbio, China) was added to each well at different time points (0, 24, 48, 72 h), and then cells were incubated at 37 °C for 2 h. The optical density (OD) value was measured at 450 nm using a microplate reader (BioTek, US).

### Wound-healing assay

The medium was removed and replaced by FBS-free culture medium, and the cells were treated with 1 μg/mL mitomycin C (Sigma, US) for 1 h. The confluent cell monolayer was scraped with a 200 μL pipette tip to make a cell-free area. Cell migration distance was measured, and photographs were taken using an optical microscope (IX53, Olympus, Japan) at 0 and 24 h after scratching.

### Transwell invasion assay

Cell invasion assay was conducted using 24-well transwell plates (LABSELECT, China) precoated with 40 μL Matrigel (Corning Incorporated, US). The transfected cells were trypsinized and washed twice with PBS, then resuspended in FBS-free medium. Cell suspension (200 μL) were added into the upper chamber (3 × 10^4^ cells/well), and 800 μL of culture medium supplemented with 10% FBS was added to the lower chamber. Before the detection, the transwell inserts were washed twice with PBS, and fixed with 4% paraformaldehyde for 20 min, then stained with 0.5% crystal violet. The number of invaded cells was counted under an optical microscope.

### Colony formation assay

The stably transfected cells were plated into 60-mm culture dishes (300 cells/dish) and placed in an incubator with 5% CO_2_ at 37 ℃. After 2 weeks, the cell colonies were washed twice with PBS and fixed with 4% paraformaldehyde for 25 min at room temperature. Colonies were then stained with Wright-Giemsa Stain (KeyGEN, China) and photographed.

### Immunohistochemistry assay

Following deparaffinization and rehydration, the paraffin-embedded tumor tissues were cut into 5-μm slices. Sections were incubated in sodium citrate buffer and heated for 10 min to retrieve antigen, then incubated with hydrogen peroxide to eliminate the activity of endogenous peroxidase. After blocking with 1% BSA (Sangon, China), the slides were incubated with primary antibody PCNA (1:100, A0264, ABclonal, China) at 4 °C overnight. The sections were then stained with HRP-conjugated goat anti-rabbit IgG (1:500, Thermo Scientific, US) for 1 h. Finally, the slides were washed, incubated with diaminobenzidine and counterstained with hematoxylin (Solarbio, China). Positive labelling was visualized and images were taken by an optical microscope.

### RNA isolation and qRT-PCR

Total RNA was isolated from cells and tissues using TRIpure (BioTeke, China). NANO 2000 spectrophotometer (Thermo Scientific, US) detected the concentration of RNA. The RNA was reverse-transcribed into cDNA using BeyoRT II M-MLV reverse transcriptase (BeyotimeBiotech, China). RT-PCR was conducted using SYBR Green I (Solarbio, China) on an Exicycler 96 RT-PCR instrument (Bioneer, Korea) according to the manufacturer's instructions. Data were analyzed by 2^−ΔΔCt^ method and GAPDH was used as a loading control. Details of the primer sequences were shown in Table 1.

**Table 1:** Primer sequences used for RT-PCR

Gene	Forward (5′–3′)	Reverse (5′–3′)
DLC1	CAGGAACCCACAGATAACC	TTCATAATCAGCAGCACCA
GATA6	GCCCAGACCACTTGCTATGA	AGCCCATCTTGACCCGAAT
CCL2	TCATAGCAGCCACCTTCATT	TCACAGCTTCTTTGGGACAC
SLFN11	TTGTCCACGGCTTACCT	TTGCCTTCCCATACCAG
RUNX3	ACCTTCGCTTCGTGGGC	TGGCTTGTGGTGCTGAGTG
SPOCK2	GACGCCAAGGGGCTCAAGGA	GGTGGGTCGGACGAGGGAAC
SPRED1	GAATGGCTGTGCTGACT	GCTCTTCGGATACCTCT

### Western blot analysis

Lysates from cells were made using RIPA (Solarbio, China) with PMSF (1 mM; Solarbio, China). Proteins were separated by SDS-PAGE. Following electrophoresis, proteins were transferred on PVDF membranes (Millipore, US). The membranes were blocked in 5% (w/v) skim milk (Sangon, China) for 1 h and then probed overnight with the appropriate primary antibodies at 4 °C. HRP-conjugated anti-IgG (goat anti-rabbit or goat anti-mouse, 1:10,000; Solarbio, China) was used as the secondary antibody. Target proteins were visualized with enhanced chemiluminescence (ECL) Plus reagent (Solarbio, China) and then images were captured. GAPDH was used as the endogenous control. The primary antibodies are as follows: EZH2 (1:1000; 5246, CST, US), H3K27me3 (1:1000; 35861, CST, US), SPOCK2 (1:500; ab217044, Abcam, UK), SPRED1 (1:500; ab77079, Abcam, UK), GAPDH (1:10000; ab8245, Abcam, UK).

### Luciferase reporter assay

A549 cells were seeded in a 12-well plate and transfected with pGL3-basic or the PGL3-SPOCK2-promoter or PGL3-SPRED1-promoter, and EZH2 overexpression plasmid using Lipofectamine 3000 (Invitrogen, USA), then treated with or without tazemetostat (2 μM). After 48 h, the cells were washed and lysed with 250 μL lysis buffer. Luciferase activity of Firefly and Renilla was measured by the Dual Luciferase Reporter Gene Assay Kit (KeyGEN, China) according to the manufacturer’s instructions.

### Statistics

Statistical analysis was carried out with using GraphPad Prism 7.0 (GraphPad Software, US). Results are represented as the mean ± standard deviation (SD). Analysis of differences were performed by unpaired *t* test, one-way ANOVA and two-way ANOVA tests. For all analyses, a *P* value < 0.05 was considered to denote statistical significance.

## Results

### Regulation of EZH2 on downstream targets

**Fig. 1:**
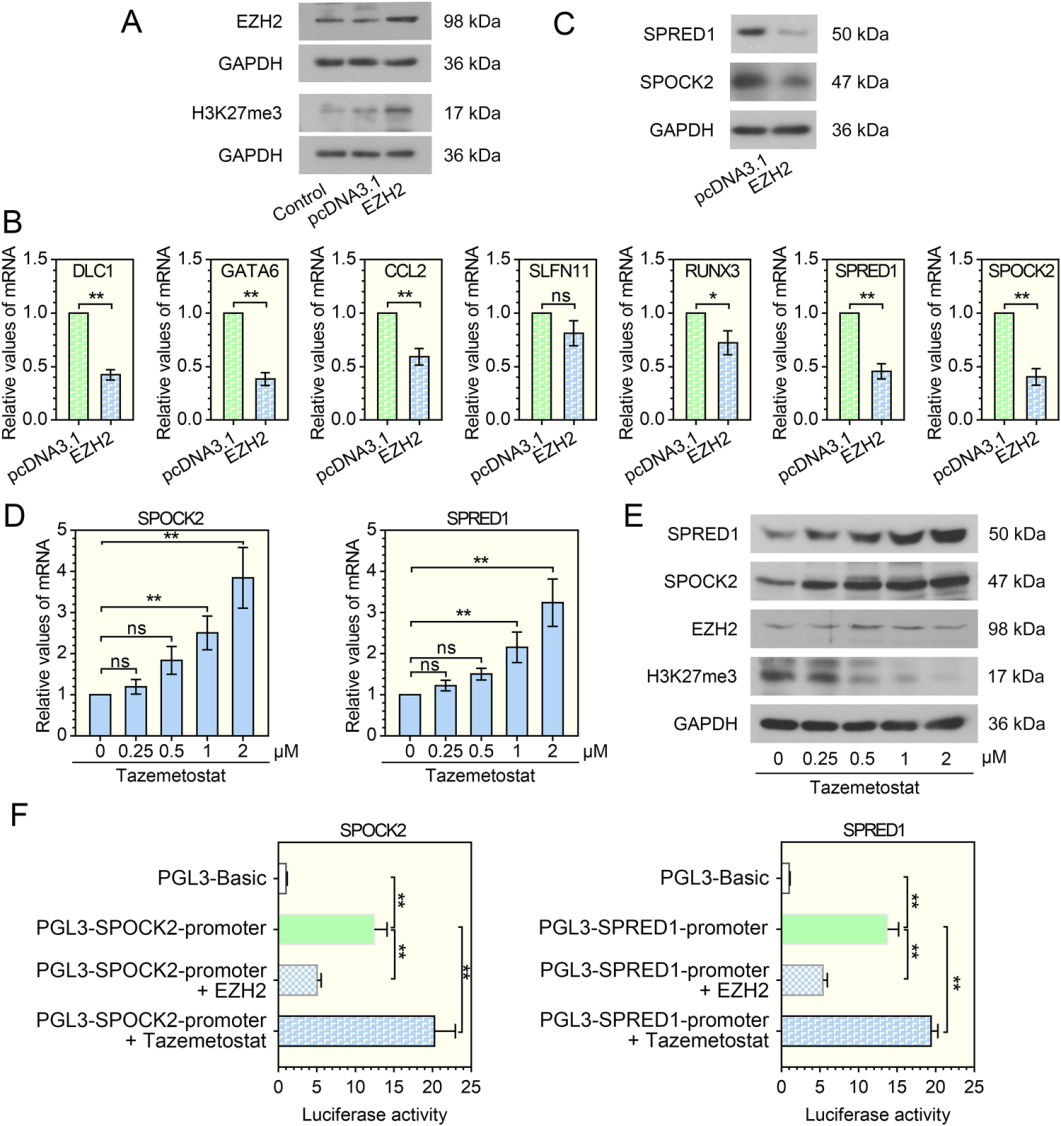
Screening of downstream targets of EZH2. EZH2 overexpression vector and its negative control vector were constructed and transfected into A549 cells. **A** Representative western blot showing EZH2 and H3K27me3 expression levels in A549 cells. *n* = 3. **B** The relative mRNA values of DLC1, GATA6, CCL2, SLFN11, RUNX3, SPRED1, and SPOCK2 were evaluated by qRT-PCR. *P < 0.05, **P < 0.01 vs the pcDNA3.1 group; *t* test; *n* = 3, repeated three times. **C** The western blot analysis of SPRED1 and SPOCK2. *n* = 3. A549 cells were treated with different concentrations (0, 0.25, 0.5, 1, 2 μM) of tazemetostat (EZH2 inhibitor) for 72 h, and then preformed **D** qRT-PCR analysis for SPOCK2 and SPRED1. **P < 0.01 vs the 0 μM tazemetostat group; one-way ANOVA*; n* = 3, repeated three times. **E** Western blot to evaluate the expression of SPRED1, SPOCK2, EZH2 and H3K27me3 upon treatment with tazemetostat. *n* = 3. **F** A549 cells were transfected with pGL3-basic or the PGL3-SPOCK2-promoter or PGL3-SPRED1-promoter, and EZH2 overexpression plasmid, then treated with or without 2 μM of tazemetostat. After 48 h, the cells were collected to measure the luciferase activity of SPOCK2 and SPRED1. **P < 0.01 vs the PGL3-Basic group, PGL3-SPOCK2-promoter group, or PGL3-SPRED1-promoter group; one-way ANOVA*; n* = 3. All data are represented as mean ± SD

We first examined the regulatory relationship between EZH2 and downstream targets. As a repressive epigenetic mark, H3K27me3 mediates the transcriptional repression of many genes [16]. Western blot analysis showed that the expression of EZH2 and H3K27me3 was increased in A549 cells after transfection with the EZH2 overexpression plasmid (Fig. 1A). Then, the mRNA levels of DLC1, GATA6, CCL2, RUNX3, SPRED1, and SPOCK2 were markedly downregulated by EZH2 overexpression (Fig. 1B), but no significant difference was observed in SLFN11. Since the expression of SPOCK2 and SPRED1 was significantly inhibited by EZH2 and their functional roles in LUAD had not been investigated, they were selected as EZH2 downstream factors for further study in this work. We further verified the regulatory effect of EZH2 on SPRED1 and SPOCK2 by western blotting (Fig. 1C), and found that EZH2 overexpression decreased the expression of SPRED1 and SPOCK2. In contrast, SPOCK2 and SPRED1 mRNA expression were significantly increased when cells were treated with 1 μM and 2 μM tazemetostat (inhibitor of EZH2, Fig. 1D). Consistently, tazemetostat treatment enhanced the protein expression of SPRED1 and SPOCK2 (Fig. 1E), while inhibited the H3K27me3 expression. Interesting, EZH2 expression was not altered by tazemetostat. We then cloned the promoter of SPOCK2 or SPRED1 into the upstream of firefly luciferase-coding region and examined the luciferase activity (Fig. 1F). Overexpression of EZH2 inhibited the activity of SPOCK2 and SPRED1 transcription, while tazemetostat activated their transcription. These findings indicated that EZH2 may suppressed the expression of SPOCK2 and SPRED1 through H3K27me3-mediated transcriptional repression.

### SPOCK2 and SPRED1 is decreased in LUAD and inhibit the proliferation of LUAD cells

**Fig. 2:**
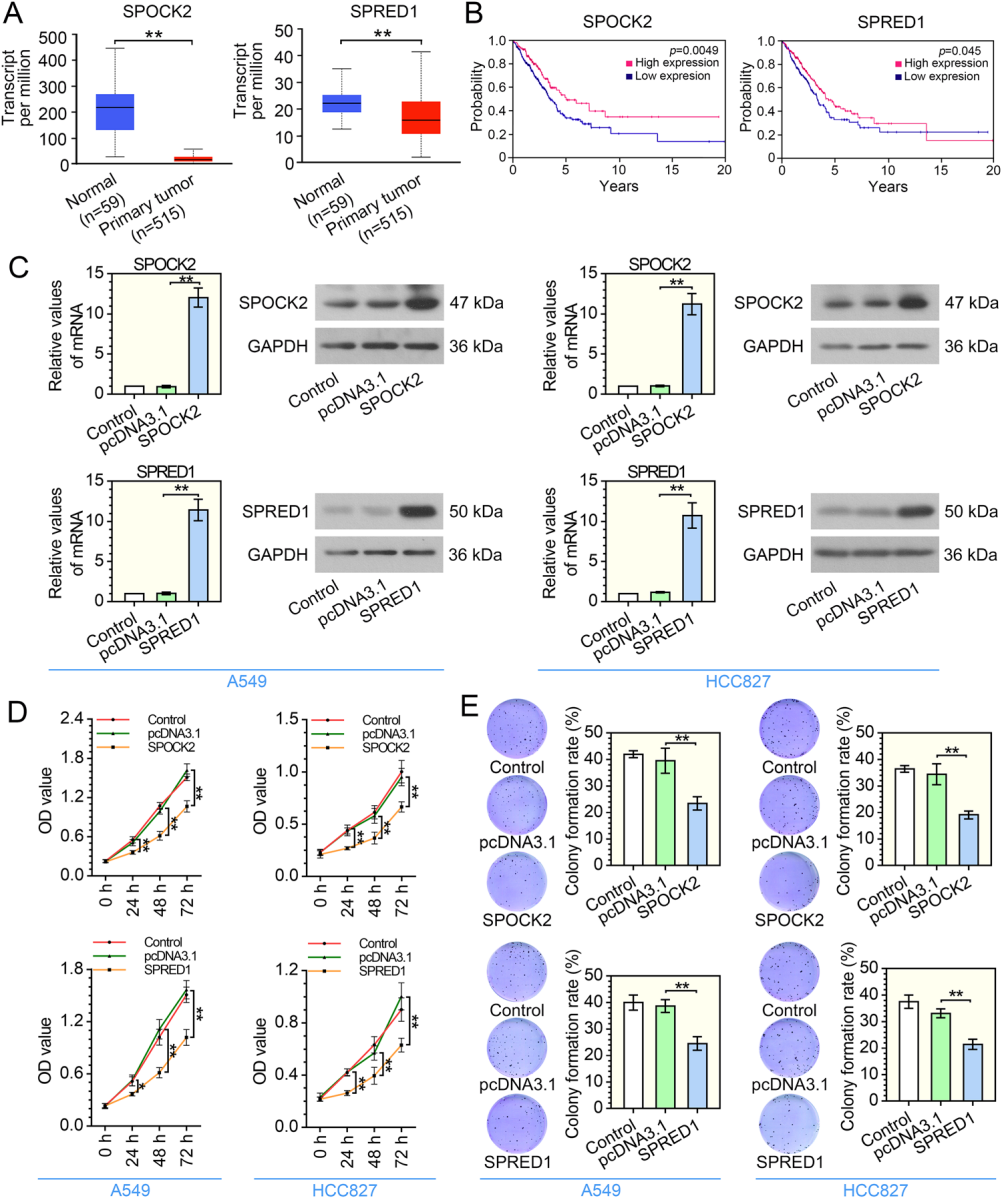
SPOCK2 and SPRED1 are decreased in LUAD cells and inhibit the proliferation of LUAD cells. **A** The expression of SPOCK2 and SPRED1 between tumor tissues and normal tissues were analyzed using TCGA LUAD database. **P < 0.01 vs the normal group; *t* test; *n* = 59/515 (normal/tumor). **B** Survival analysis of SPOCK2 and SPRED1 in TCGA LUAD database. The best high and low expression cut-off values were 9.68 for SPOCK2 and 7.73 for SPRED1. SPOCK2 or SPRED1 overexpression vector and the control vector were transfected into A549 and HCC827 cells respectively, and the stable transfected cell lines were screened with G418. **C** The expression of SPOCK2 and SPRED1 in LUAD cells were analyzed by qRT-PCR and western blot. **P < 0.01 vs the pcDNA3.1 group; one-way ANOVA; *n* = 3, repeated three times. **D** CCK-8 assay was performed to evaluate LUAD cell proliferation. *P < 0.05, **P < 0.01 vs the pcDNA3.1 group; two-way ANOVA; *n* = 3, repeated five times. **E** The effect of overexpression of SPOCK2 or SPRED1 on colony formation of LUAD cell. **P < 0.01 vs the pcDNA3.1 group; one-way ANOVA; *n* = 3. All data are represented as mean ± SD

Next, we evaluated the expression of SPOCK2 and SPRED1 in the LUAD samples from the TCGA database, and found that the expression of SPOCK2 and SPRED1 in the tumor tissues of LUAD patients was significantly decreased (Fig. 2A). Moreover, the prognostic values of SPOCK2 and SPRED1 in LUAD were analyzed. We found that a high SPOCK2 or SPRED1 expression level correlated with better survival in LUAD (Fig. 2B). Subsequently, the expression efficiencies of SPOCK2 and SPRED1 were determined by qRT-PCR and western blot analysis (Fig. 2C). Compared with the pcDNA3.1 group, SPOCK2 expression was significantly increased in the SPOCK2 overexpression group, and the same result was also obtained in the SPRED1 overexpression group. Afterward, we studied the effect of SPOCK2 and SPRED1 on LUAD cell proliferation. CCK-8 assay showed that the overexpression of SPOCK2 or SPRED1 led to a marked decrease in the viability of A549 and HCC827 cells (Fig. 2D). In colony formation assay (Fig. 2E), the overexpression of SPOCK2 or SPRED1 showed a decreased proliferation ability, as indicated by their low colony formation rates. These data suggest that SPOCK2 and SPRED1 expression are decreased in LUAD, and overexpression of SPOCK2 or SPRED1 inhibits LUAD cell proliferation.

### Overexpression of SPOCK2 or SPRED1 suppresses migration ratio and invasion activity in LUAD cells

**Fig. 3:**
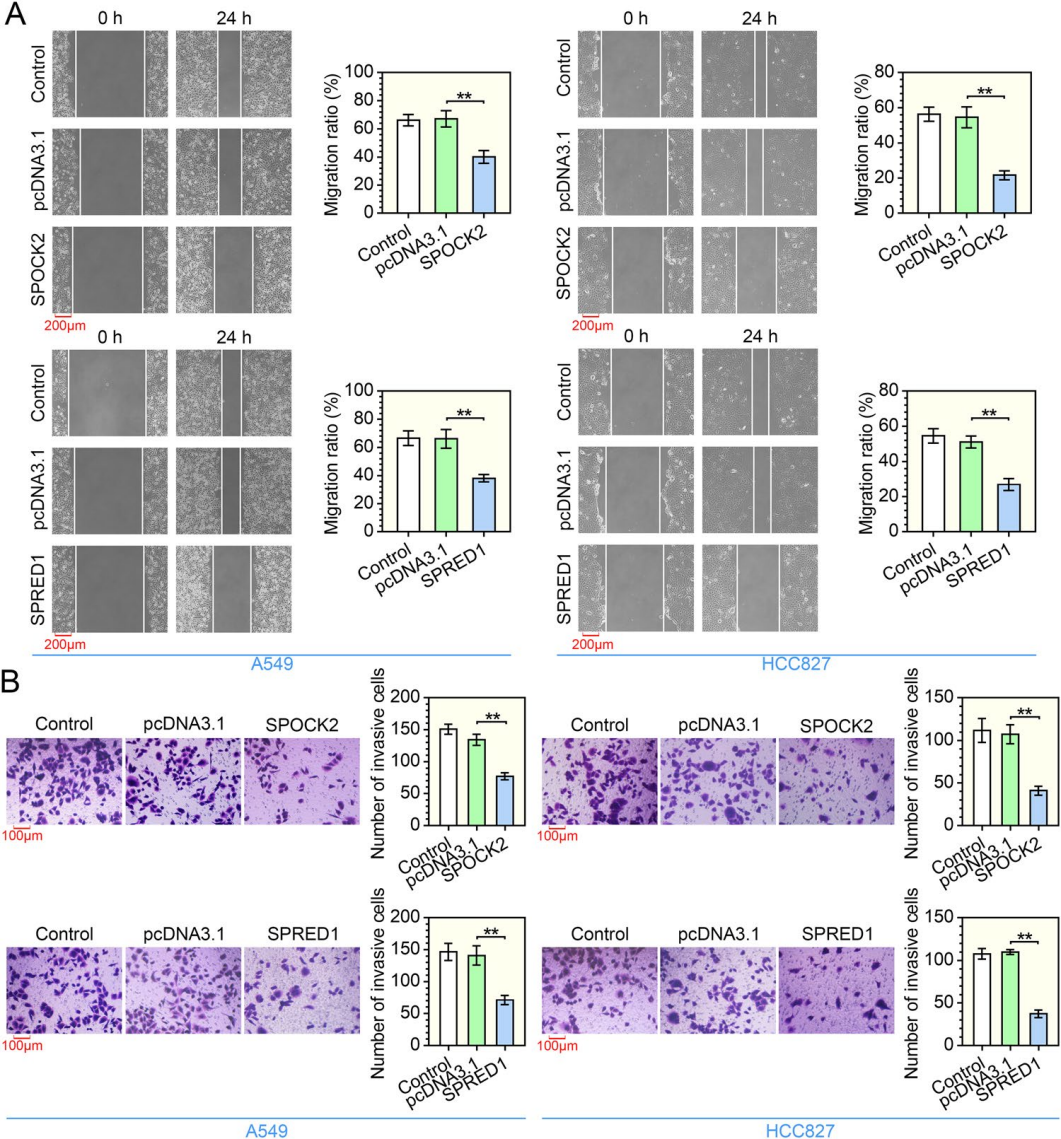
Overexpression of SPOCK2 or SPRED1 suppresses the migration ratio and invasion activity in LUAD cells. **A** The migration ratio of A549 and HCC827 cells transfected with SPOCK2 or SPRED1 overexpression plasmid was assessed by wound-healing assay. **P < 0.01 vs the pcDNA3.1 group; one-way ANOVA; *n* = 3; magnification, 100×. **B** The invasion ability of LUAD cells was evaluated by the transwell assay. **P < 0.01 vs the pcDNA3.1 group; one-way ANOVA; *n* = 3; magnification, 200×. All data are represented as mean ± SD

To examine the role of SPOCK2 and SPRED1 on LUAD cell migration, we carried out wound-healing assay. A549 and HCC827 cells overexpressing SPOCK2 or SPRED1 exhibited decreased cell migration ratio (Fig. 3A). Moreover, we examined cell invasion activity by transwell assay. Consistent with wound-healing assay, overexpression of SPOCK2 or SPRED1 reduced the number of invasive A549 and HCC827 cells (Fig. 3B). These results indicate that overexpression of SPOCK2 or SPRED1 exhibits the inhibitory efficacy on migration ratio and invasion activity of LUAD cells.

### The antineoplastic effects of SPOCK2 or SPRED1 are impaired by EZH2

**Fig. 4:**
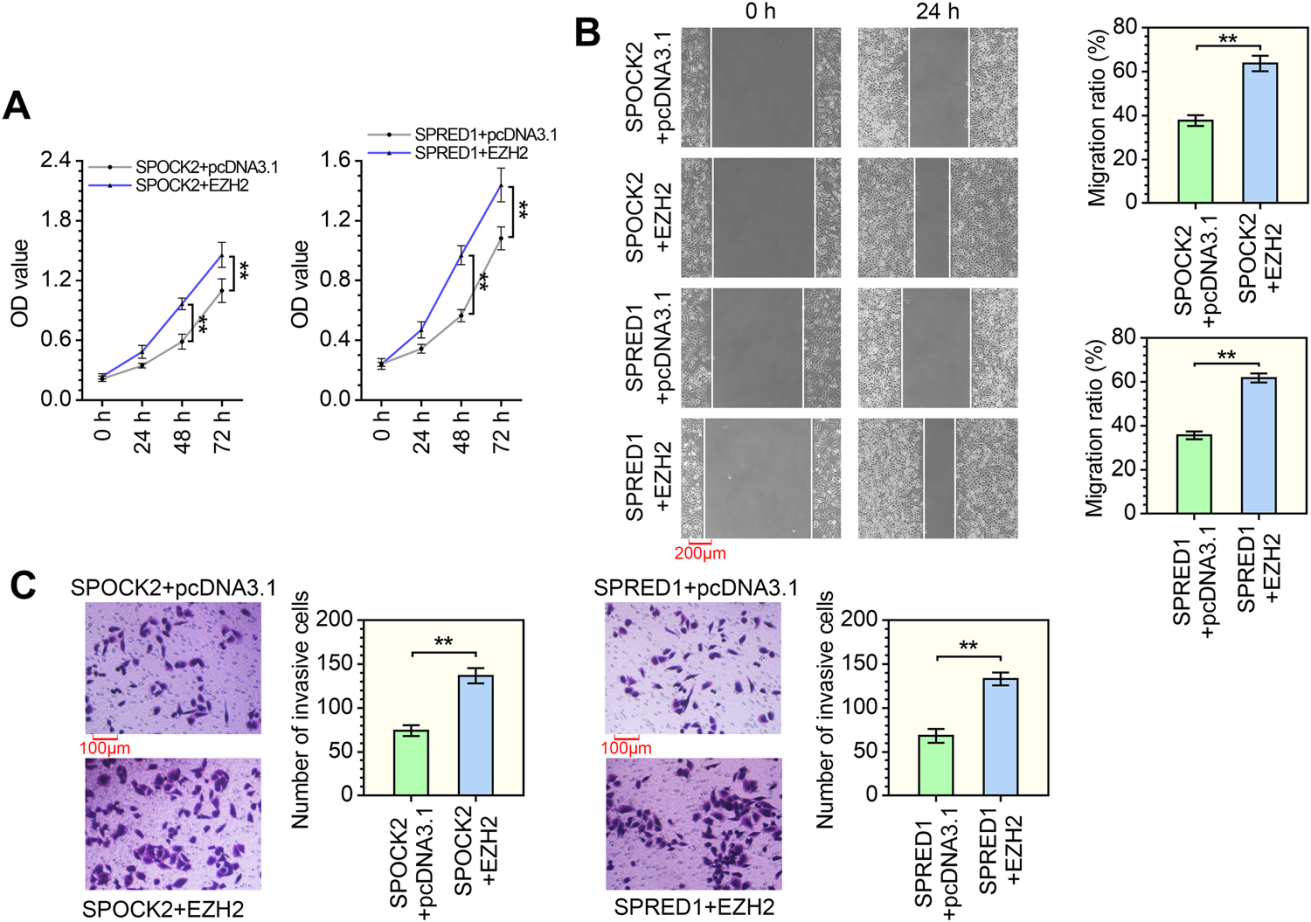
The antineoplastic effects of SPOCK2 or SPRED1 are impaired by EZH2. A549 cells were transfected with SPOCK2 or SPRED1 overexpression vector and/or the control vector or EZH2 overexpression vector. **A** CCK-8 assay was performed to evaluate cell proliferation. **P < 0.01 vs the SPOCK2 + pcDNA3.1 group or SPRED1 + pcDNA3.1 group; two-way ANOVA; *n* = 3, repeated five times. **B** The migration ratio was evaluated by wound-healing assay. **P < 0.01 vs the SPOCK2 + pcDNA3.1 group or SPRED1 + pcDNA3.1 group; *t* test; *n* = 3; magnification, 100×. **C** The invasion ability was tested by the transwell assay. **P < 0.01 vs the SPOCK2 + pcDNA3.1 group or SPRED1 + pcDNA3.1 group; *t* test; *n* = 3; magnification, 200×. All data are represented as mean ± SD

We examined whether the antineoplastic effects of SPOCK2 or SPRED1 on LUAD cells are inhibited by EZH2. As shown in Fig. 4A, the CCK-8 assay performed in A549 cells showed that EZH2 overexpression significantly increased the proliferation of cells overexpressing SPOCK2 or SPRED1. Furthermore, EZH2 overexpression led to an enhanced migration ratio of SPOCK2 or SPRED1 upregulated cells (Fig. 4B). Similarly, the transwell assay revealed that the ability of invasion was significantly increased in SPOCK2- or SPRED1-overexpressed cells following the upregulation of EZH2 (Fig. 4C). The above data strongly imply that EZH2 inhibits the effects of SPOCK2 and SPRED1 on proliferation, migration and invasion of LUAD cells.

### Overexpression of SPOCK2 or SPRED1 restrains tumor growth of LUAD cells in vivo

**Fig. 5:**
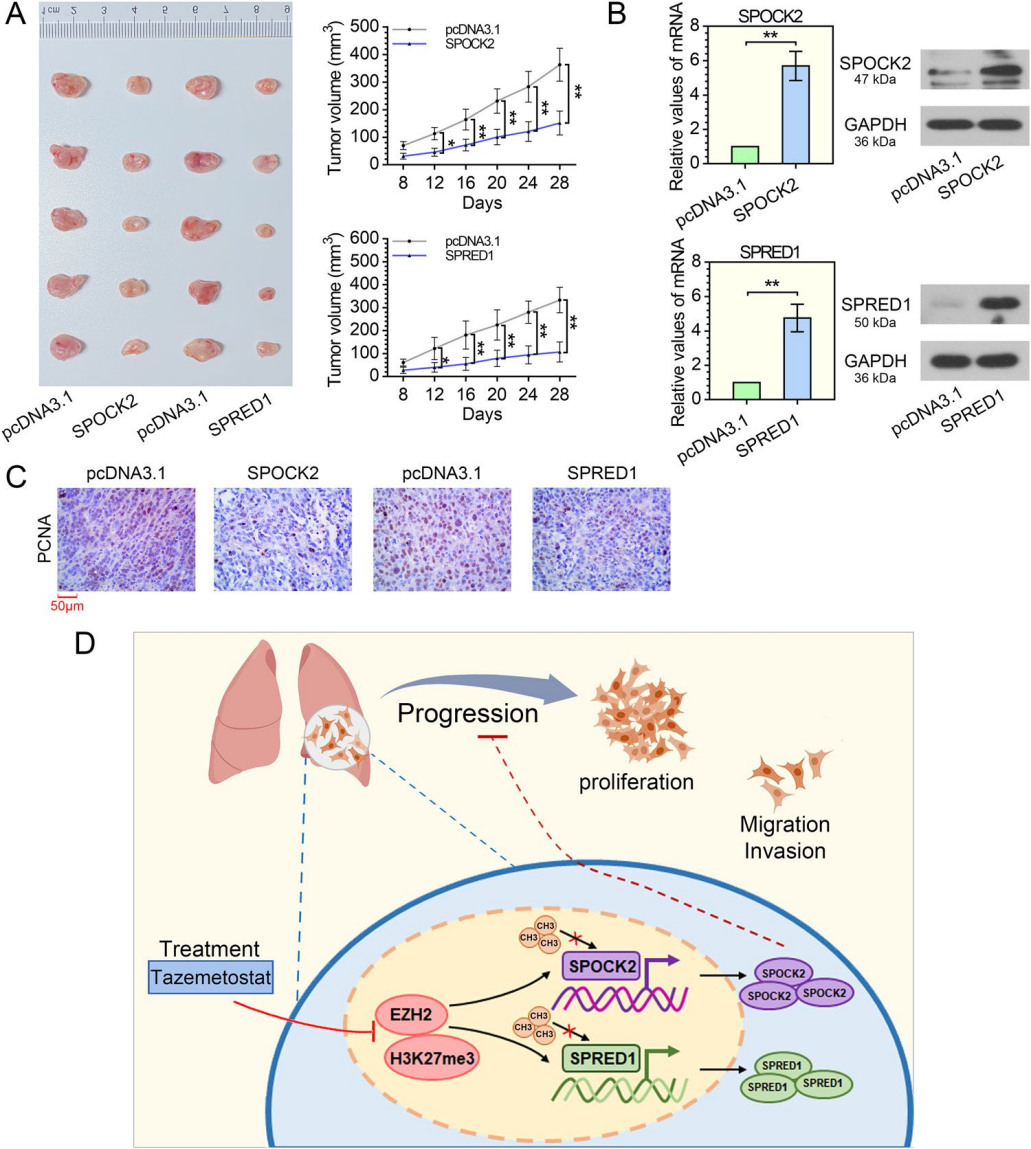
Overexpression of SPOCK2 or SPRED1 restrains LUAD cell growth in vivo. Nude mice were injected subcutaneously with 2 × 10^6^ of stable transfected A549 cells to construct a xenograft tumor model. **A** Representative pictures and the tumor volume curves of A549 xenograft tumors. *P < 0.05, **P < 0.01 vs the pcDNA3.1 group; two-way ANOVA; *n* = 5. **B** The qRT-PCR and western blot analysis of SPOCK2 and SPRED1 in A549 tumors. **P < 0.01 vs the pcDNA3.1 group; t-test; *n* = 5, repeated three times. **C** Typical images of PCNA immunohistochemistry staining. *n* = 5; magnification, 400×. **D** Graphical summary of the effects of EZH2, SPOCK2, and SPRED1 in LUAD: EZH2-mediated H3K27me3 transcription repressed SPOCK2 and SPRED1, and suppressed the expression of SPOCK2 and SPRED1 in tumor cells of LUAD, thereby facilitating the proliferation, migration, and invasion of LUAD cells. All data are represented as mean ± SD

To elucidate the effect of SPOCK2 or SPRED1 on tumor growth of LUAD cells in vivo, we subcutaneously injected the SPOCK2 or SPRED1 overexpressed A549 cells into the nude mice. As shown in Fig. 5A, overexpression of SPOCK2 or SPRED1 significantly restrained tumor growth. Moreover, qRT-PCR and western blot analysis revealed that SPOCK2 and SPRED1 were highly upregulated in those xenografts that grew from A549 cells with SPOCK2 or SPRED1 overexpression (Fig. 5B). Immunohistochemical analysis confirmed that overexpression of SPOCK2 or SPRED1 could reduce the expression of PCNA in xenografts developed from A549 cells (Fig. 5C). Taken together, the above results demonstrate that SPOCK2 or SPRED1 might retard tumor cell growth in vivo by restraining cell proliferation.

## Discussion

LUAD, as a predominant cause of mortality, is aggressive and manifested with rapid proliferation and migration [17]. However, effective targeting therapeutic approaches are still lacking. Here, we found that EZH2-mediated inhibition of multiple downstream genes, especially SPOCK2 and SPRED1. Meanwhile, we confirmed that SPOCK2 and SPRED1 were markedly downregulated in LUAD patients, and expression of SPOCK2 or SPRED1 was positively correlated with a favorable prognosis. Further analyses showed that the overexpression of SPOCK2 or that of SPRED1 statistically inhibited LUAD cells proliferation, migration ratio, invasion activity, and tumor growth. Furthermore, the inhibition of SPOCK2 or SPRED1 on the malignant phenotypes of LUAD was abolished by EZH2 upregulation (Fig. 5D). Thus, current work provides new insights into understanding the potential roles of EZH2-mediated SPOCK2 or SPRED1 in LUAD and their potential as biomarkers for cancer therapy and prognosis.

The SPOCK family includes three proteins, namely SPOCK1, SPOCK2 and SPOCK3. SPOCK2 is synthesized by lung epithelial cells and fibroblasts and localized in the extracellular matrix, which plays significant roles in limiting the malignant behavior of cancer cells [18]. SPOCK2 was identified as bronchopulmonary dysplasia susceptibility gene and had important implications for lung development [19]. In 2008, a study first showed a link between the SPOCK2 gene and cancer [20]. In recent years, a growing number of studies showed that SPOCK2 played significant roles in many cancers. Specifically, SPOCK2 was highly expressed in ovarian cancer [21], and high level of SPOCK2 promoted migration and invasion of glioma cells [22], whereas more evidences suggested a tumor suppressor role for SPOCK2. SPOCK2 expression was downregulated in prostate cancer and its overexpression suppressed prostate cancer cell invasion and migration [18]. Similarly, SPOCK2 expression was detected in normal endometrium, while its expression was decreased in endometrial cancer. In addition, SPOCK2 upregulation impaired the ability of invasive and adherence, and increased apoptosis of endometrial cancer cells, its overexpression suppressed cell proliferation via impeding cell cycle progression [23]. Notably, in a study of LUAD, the transcriptional and proteomic levels of SPOCK2 were found to reduce in LUAD tissues, by bioinformatics analysis, and was positive associated with OS of LUAD patients, indicating SPOCK2 may be a promising prognostic biomarker for LUAD [24]. In this study, expression levels of SPOCK2 in LUAD patient tumor tissues were remarkable lower than normal lung tissues, and high level of SPOCK2 expression predicted better survival. Furthermore, the overexpression of SPOCK2 inhibited the malignant phenotypes of LUAD. Overall, our results demonstrate the inhibitory role of SPOCK2 on LUAD. On the other hand, SPOCK2 expression was found to elevate in EZH2 knockout cells [25]. Sambuudash et al. found that methylation level of SPOCK2 in colon cancer was higher than that in adjacent normal mucosa [26]. Another study reported that epigenetic inactivation of SPOCK2 caused by SPOCK2 hypermethylation, may be associated with the exacerbation of ovarian endometriosis [27]. Consistently, our findings revealed that the transcriptional repression of EZH2-mediated H3K27me3 on SPOCK2 may be a mechanism for tumorigenesis and progression of LUAD.

The SPRED family includes SPRED1, SPRED2 and SPRED3. SPRED1 and SPRED2 are structurally similar in that they both contain an N-terminal EVH-1 domain, a central c-KIT–binding domain (KBD) and a C-terminal SPR domain [28]. SPRED1 has been implicated in several biological process, such as signal transduction, dysplasia and tumor development [29]. In osteosarcoma cells, SPRED1 inhibited cell proliferation and migration by suppressing stress fiber formation [30]. SPRED1 negatively regulated signaling pathways contributing to hepatic carcinoma progression, and its overexpression inhibited the malignant features of hepatoma cells [31]. Ablain et al. reported that SPRED1 was loss in human cutaneous melanoma, and low expression of SPRED1 was correlated with poor prognosis of patients with melanoma [32]. Additionally, a recent study demonstrated that SPRED1 deletion was able to augment drug resistance in chronic myeloid leukemia [33]. These lines of evidence suggest that SPRED1 may be a tumor suppressor. In the current study, a significant decrease in SPRED1 expression was observed in LUAD tissues, and a high level of SPRED1 expression favored better survival in LUAD. Upregulation of this gene can inhibit cell proliferation, migration ratio, invasion activity, and tumor growth. The results demonstrate the critical role of SPRED1 in impeding malignant progression of LUAD. Moreover, a recent study indicated that SPRED1 expression was upregulated by knockdown of EZH2 in endometrial adenocarcinoma cells [12]. Consistent with this finding, we confirmed in our study that EZH2, as the upstream regulator of SPRED1, negatively regulate SPRED1 expression by inhibiting its transcriptional activity in LUAD cells.

In addition, we also demonstrated that treatment of tazemetostat enhanced the transcriptional activity of SPOCK2 and SPRED1 and upregulated their expression, although it did not affect the expression of EZH2. Tazemetostat has a unique mechanism of action, it can inhibit the activity of EZH2, but the expression of EZH2 will not be affected [8, 34]. Consequently, our findings suggest drug interference with EZH2 activity may as a promising strategy to increase SPOCK2 and SPRED1 expression levels in LUAD, thus inhibiting the progression of LUAD. However, the specific mechanism of action needs to be further explored.

Collectively, our data provide the evidence that SPOCK2 and SPRED1 are epigenetic inhibited by EZH2, and play important roles in inhibiting proliferation, migration and invasion of LUAD cells. The findings provide a potential basis for future development of lung cancer treatments. However, the current study also has two major limitations. For one, affected by novel coronavirus pneumonia, tissue samples from LUAD patients are difficult to collect, and collected samples were rarely sufficient for examination. Therefore, the present study lacks support from the results of actual clinical samples. We intend to continue collecting human samples to validate the expression of SPOCK2 and SPRED1 in tumor tissues. For another, it was insufficient to use only two cell lines, A549 and HCC827, for investigating the functions of SPOCK2 and SPRED1 in LUAD. We will thus further test it in more LUAD cell lines.

## Data Availability

All data generated during the current study are included in the article.
